# The matricellular protein CYR61 interferes with normal pancreatic islets architecture and promotes pancreatic neuroendocrine tumor progression

**DOI:** 10.18632/oncotarget.6411

**Published:** 2015-11-27

**Authors:** Yu-Ting Huang, Qiang Lan, Lionel Ponsonnet, Marisa Blanquet, Gerhard Christofori, Jelena Zaric, Curzio Rüegg

**Affiliations:** ^1^ Department of Medicine, Faculty of Science, University of Fribourg, Fribourg, Switzerland; ^2^ National Center for Competence in Research (NCCR), Molecular Oncology, Swiss Institute for Experimental Cancer Research (ISREC)-Ecole Polytechnique Fédérale de Lausanne (EPFL), Lausanne, Switzerland; ^3^ Department of Biomedicine, University of Basel, Basel, Switzerland

**Keywords:** CYR61, transgenic model, angiogenesis, invasion, insulinoma

## Abstract

The significance of matricellular proteins during development and cancer progression is widely recognized. However, how these proteins actively contribute to physiological development and pathological cancer progression is only partially elucidated. In this study, we investigated the role of the matricellular protein Cysteine-rich 61 (CYR61) in pancreatic islet development and carcinogenesis. Transgenic expression of CYR61 in β cells (Rip1CYR mice) caused irregular islets morphology and distorted sorting of α cells, but did not alter islets size, number or vascularization. To investigate the function of CYR61 during carcinogenesis, we crossed Rip1CYR mice with Rip1Tag2 mice, a well-established model of β cell carcinogenesis. Beta tumors in Rip1Tag2CYR mice were larger, more invasive and more vascularized compared to tumors in Rip1Tag2 mice. The effect of CYR61 on angiogenesis was fully abrogated by treating mice with the anti-VEGFR2 mAb DC101. Results from *in vitro* assays demonstrated that CYR61 modulated integrin α_6_β_1_-dependent invasion and adhesion without altering its expression. Taken together, these results show that CYR61 expression in pancreatic β cells interferes with normal islet architecture, promotes islet tumor growth, invasion and VEGF/VERGFR-2-dependent tumor angiogenesis. Taken together, these observations demonstrate that CYR61 acts as a tumor-promoting gene in pancreatic neuroendocrine tumors.

## INTRODUCTION

In recent years, focus of cancer research has expanded from cell autonomous phenomena to the complex interaction network between tumor microenvironment and cancer cells. Tumor microenvironment comprises stromal cells, soluble factors, extracellular matrix and matricellular proteins [1]. While matrix proteins constitute the physical framework of the tissue and provide mechanical modulation of cellular activities, cells can also secrete matricellular proteins into the extracellular space. Through binding with both membrane protein receptors and the extracellular matrix, matricellular proteins actively participate in the regulation of cell-cell and cell-matrix interactions. Several families of matricellular proteins have been identified and their functions are gradually unveiled [2].

Cysteine-rich 61 (CYR61) belongs to the CCN (CYR61, CTGF and NOV) family of secreted matricellular proteins. It is composed of an N-terminal signal peptide for secretion, four conserved domains homologous to insulin-like growth factor-binding protein (IGFBP), von Willebrand factor type C repeats (VWC), thrompospondin type 1 repeat (TSP-1) and a carboxyl-terminal (CT) domain containing a cysteine knot motif, respectively [3]. The latter three domains contain binding sites for cell surface heparin sulfate proteoglycans (HSPG) and integrin adhesion receptors, including α_2_β_1_, α_6_β_1_, α_v_β_1_, α_v_β_3_ and α_v_β_5_ [4]. The capability of binding to various cell surface receptors allows CYR61 to regulate diverse cellular activities in a context-dependent manner [3, 5, 6]. For instance, CYR61 enhances adhesion and tube formation of quiescent endothelial cells through integrin α_6_β_1_, while in activated endothelial cells, the same functions are mediated by integrin α_v_β_3_ [7]. Besides cell adhesion and tube formation, CYR61 also promotes endothelial cell migration, proliferation and survival [7].

From *in vitro* and *in vivo* experiments using cell lines, CYR61 was shown to enhance cancer cell growth and invasion in breast [8, 9], gastric [10, 11], ovarian cancers [12] and glioma [13, 14]. Clinical studies on these cancers revealed positive correlations between CYR61 expression level and tumor stages, metastasis, recurrence and reduced survival [12, 15–17], suggesting a cancer-promoting function of CYR61. However, experimental and clinical observations in non-small-cell lung cancer (NSCLC) showed that CYR61 suppresses tumor growth, migration and late stage progression [18, 19]. In endometrial cancer and hepatocellular carcinoma, the function of CYR61 remains unclear since both positive and negative correlations between CYR61 level and cancer progression have been reported [20–23]. The opposing roles of CYR61 among different types and stages of cancer indicate the urge of studying this molecule in additional *in vivo* tumor models.

In this study, we sought to characterize the function of CYR61 in normal tissue development and the development of cancerous lesions from the corresponding tissue. To study its role in normal tissue development we first established a tissue-specific transgenic mouse line expressing human CYR61 in the insulin-secreting β cells of pancreatic islets of Langerhans (Rip1CYR). To study the role of CYR61 in tumor development, we crossed the Rip1CYR mice with another transgenic mice, Rip1Tag2, that spontaneously form β cell tumors (i.e. pancreatic neuroendocrine tumors, PNET) [24]. Analysis of these mice revealed that CYR61 expression in normal pancreatic β cells alters islet architecture but not growth and vasculararization, while expression in tumorigenic mice promotes islet tumor growth, angiogenesis and invasion.

## RESULTS

### Generation of Rip1CYR transgenic mice

**Figure 1:**
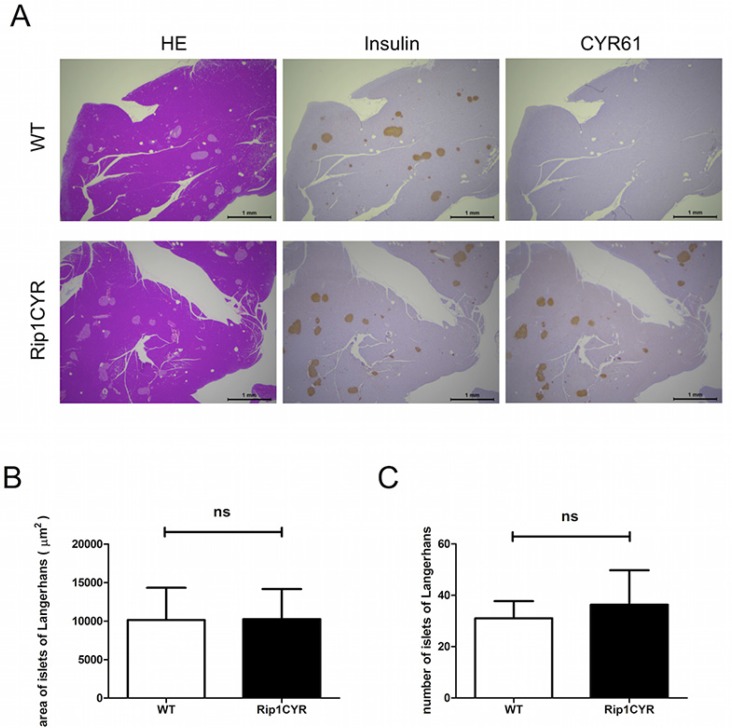
Tissue-specific expression of CYR61 does not alter the size or number of islets of Langerhans in Rip1CYR mice (**A**) Serial sections of pancreata from wild-type (WT, top row) and Rip1CYR (bottom row) mice were stained with HE, anti-insulin, anti-CYR61 antibodies (from left to right). Scale bar: 1 mm. (**B**–**C**) Ectopic expression of CYR61 did not change the size (B) or number (C) of islets of Langerhans in both wild type (WT) and Rip1CYR mice. Results represent mean values ± SD. ns: non significant.

To generate transgenic mice expressing human CYR61 in the pancreatic β cells of the islets of Langerhans, we cloned a 1620 bp cDNA fragment containing the human *CYR61* ORF (1146 bp) between the rat insulin gene II promoter and the DNA fragment containing SV40 large T and small t antigen intron and polyadenylation signal (Supplementary Figure 1A). Two transgenic lines with stable transgene expression to their progeny were obtained. Analysis of transgene genomic integration was performed by PCR on genomic DNA (Supplementary Figure 1B). Protein expression of the transgene was confirmed by western blotting using whole pancreata lysates of wild type C57Bl/6 and Rip1CYR mice (Supplementary Figure 1C, left panel). Immunohistochemical staining confirmed that CYR61 was specifically expressed in all islets of Langerhans of the Rip1CYR mice (Figure 1A).

### CYR61 alters the architecture but not the size of islets of Langerhans and distorts the segregation of α cells

**Figure 2:**
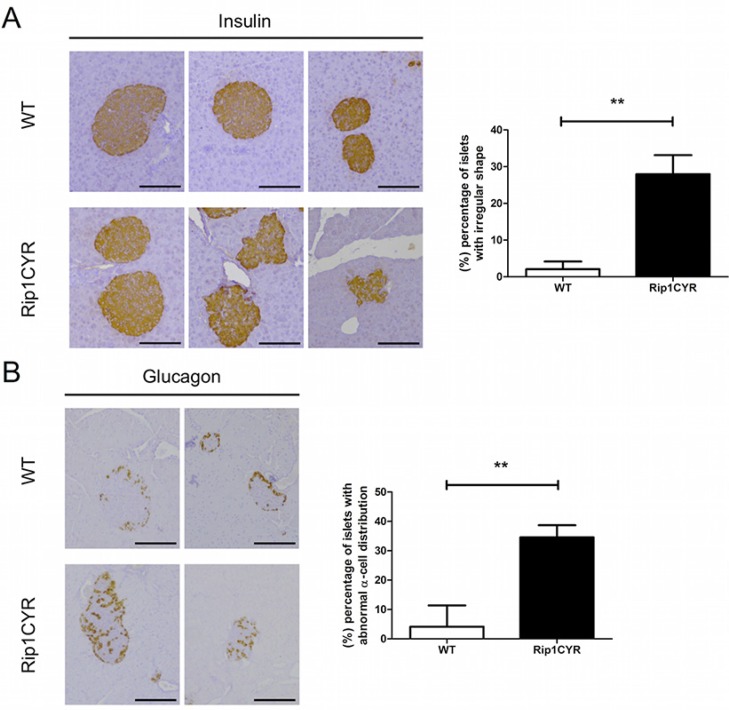
Morphological changes of islets of Langerhans and distorted segregation of α cells in Rip1CYR mice (**A**) The shape of islets, as revealed by insulin staining, was altered by transgenic expression of CYR61. Scale bar: 100 μm. Percentage of islets with irregular shape is presented by the bar graph on the right. (**B**) Glucagon staining revealed that the localization of α cells is altered in the islets of Rip1CYR mice. Scale bar: 100 μm. Percentage of islets with altered segregation of α cells is presented by the bar graph on the right. Results represent mean values ± SD. **p < 0.01.

The transgenic Rip1CYR mice bread at mendelian rate, had normal life span and did not show obvious pathologies. Although CYR61 has been shown to enhance cell proliferation, the transgene had no significant impact on either size or number of islets (Figure 1B, 1C). Besides, there was no islet tumor formed up to 18 months of age in the Rip1CYR mice examined by histological staining (data not shown). The shape of the islets in Rip1CYR mice, however, was often irregular with cells protruding into the surrounding exocrine pancreatic tissue (Figure 2A, left panel). Quantification revealed that up to 30% of the islets of Rip1CYR mice were irregularly shaped compared to only 2% in wild type animals (Figure 2A, right panel). Moreover, glucagon staining showed that the α cells were not properly segregated to the peripheral cell layers in about 1/3 of the islets of Rip1CYR mice while only less than 5% of islets showed such phenotype in wild type animals (Figure 2B).

These results indicate that ectopic expression of CYR61 in β cells does not induce hyperplastic growth or tumor formation but causes irregular shape, invasive borders and distorted segregation of the glucagon-producing α cells in about one third of the islets.

### The vasculature of Langerhans' islets in Rip1CYR mice shows no difference compared to wild type mice

**Figure 3:**
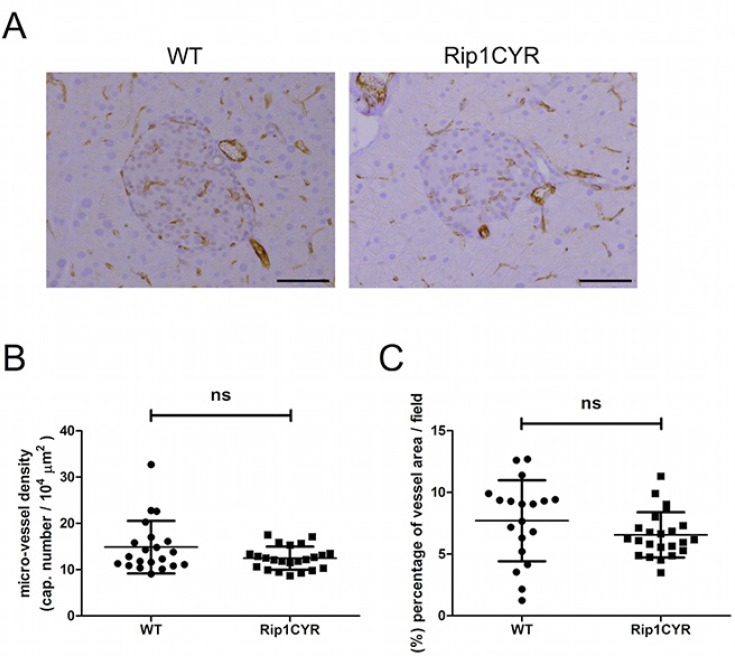
CYR61 does not affect the vasculature in the islets of Langerhans (**A**) The vasculature of islets of Langerhans in both WT and Rip1CYR mice was revealed by CD31 staining. Scale bar: 100 μm. (**B**–**C**) Micro-vessel density (B) and the area occupied by vessels (C) were not altered in Rip1CYR mice compared to WT mice. Results represent mean values ± SD. ns: non significant.

CYR61 was reported to enhance proliferation, survival, motility and tube formation of endothelial cells *in vitro* [6]. Results from *Cyr61*-null mice also demonstrated its critical role in vascular development [25]. To examine the potential effects of CYR61 expression on the development of the vasculature in pancreatic islets, we analyzed vascular parameters in wild type and Rip1CYR pancreata by CD31 staining (Figure 3A). Quantification of both micro-vessel density (Figure 3B) and the area occupied by vessels (Figure 3C) in the islets revealed no apparent difference between the two genotypes of mice. Also, the vasculatures in the exocrine part of the pancreas of both genotypes were indistinguishable (data not shown).

These findings demonstrate that the development of the vasculature in the islets and exocrine part is not affected by CYR61 expression in the insulin-producing β cells.

### The β tumors in Rip1Tag2CYR mice are larger compared to tumors in Rip1Tag2 mice

**Figure 4:**
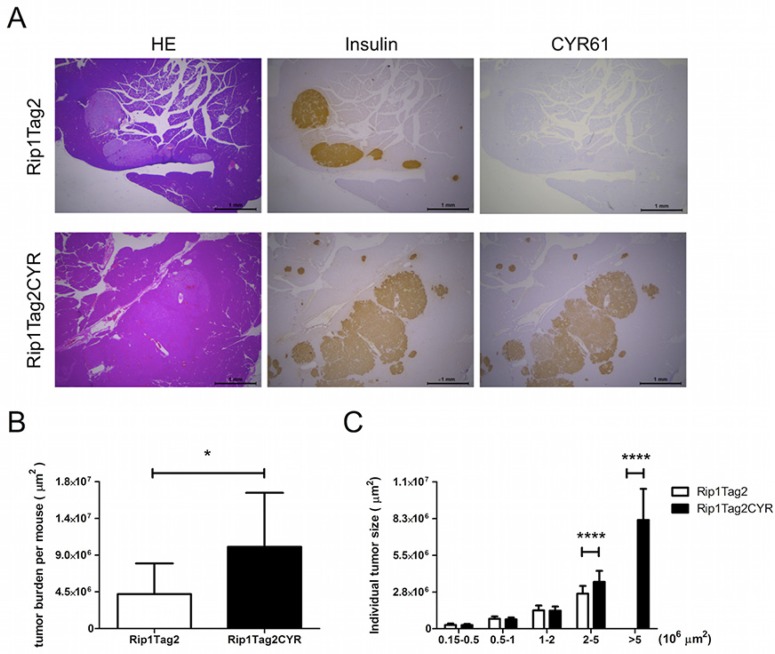
CYR61 promotes growth of β cell-derived tumors (**A**) Serial sections of pancreata from Rip1Tag2 (top row) and Rip1Tag2CYR (bottom row) mice were anti-insulin, anti-CYR61 antibodies (from left to right). Scale bar: 1 mm. (**B**) Tumor burden (total tumor area) of each mouse was quantified using Image J software. (**C**) Tumors were categorized into 5 groups according to tumor size. Tumors in the Rip1Tag2CYR mice were significantly larger. Results represent mean values ± SD. *p < 0.05; ****p < 0.0001.

In order to study the effect of CYR61 on tumor progression, we crossed Rip1CYR mice with Rip1Tag2 mice to obtain double-transgenic Rip1Tag2CYR mice expressing CYR61 in β tumors (Supplementary Figure 1B and 1C). Staining of insulin and CYR61 in pancreata from 13 week-old Rip1Tag2 and Rip1Tag2CYR revealed that expression of transgenic CYR61 was largely maintained in all β tumors (Figure 4A). Sporadic, partial loss of CYR61 or insulin expression was found in rare tumor regions, possibly due to de-differentiation during tumor progression (not shown).

Next, we measured total tumor burden of each mouse and the size of individual tumors. Analysis showed that the total tumor burden of Rip1Tag2CYR mice was approximately double compared to that of age-matched Rip1Tag2 mice (Figure 4B). By separating individual tumors into 5 groups according to size, differences were limited to the 2 groups with larger sizes (Figure 4C). In fact, tumors with a surface greater than 5*10^6^ μm^2^ were only observed in Rip1Tag2CYR mice. This result clearly demonstrates that CYR61 promotes tumor growth.

### CYR61 enhances VEGF/VEGFR2-dependent angiogenesis in β tumors in Rip1Tag2CYR mice

**Figure 5:**
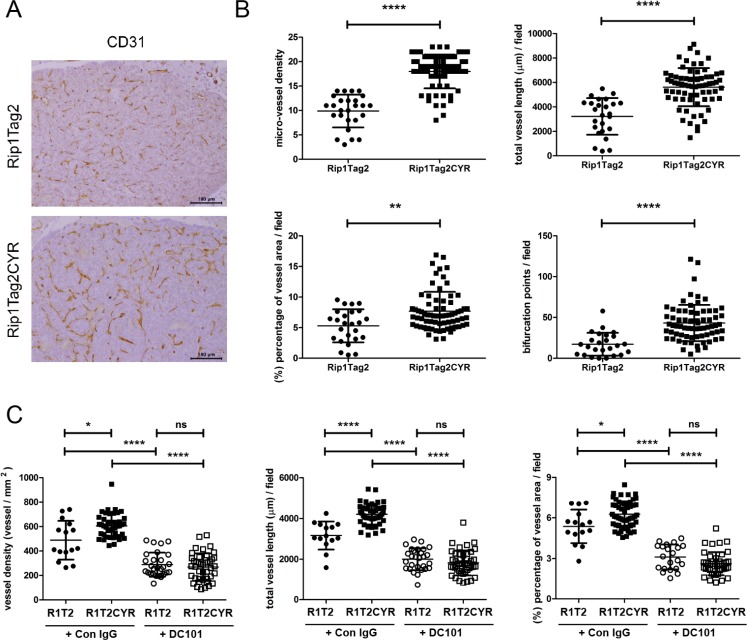
CYR61 enhances VEGF-dependent tumor angiogenesis in β cell-derived tumors (**A**) The vasculature of β tumors in Rip1Tag2 and Rip1Tag2CYR mice was revealed by CD31 staining. Scale bar: 100 μm. (**B**) Tumor angiogenesis was evaluated by 4 parameters: micro-vessel density, total vessel length, area covered by vessels and number of bifurcation points. All four parameters are increased in Rip1Tag2CYR mice compared to Rip1Tag2 mice. (**C**) Rip1Tag2 (R1T2) and Rip1Tag2CYR (R1T2CYR) mice were treated with either isotype control IgG (Con IgG) or DC101 antibodies. The vasculature of β tumors was evaluated by micro-vessel density, total vessel length and area covered by vessels. Results represent mean values ± SD. *p < 0.05; **p < 0.01; ****p < 0.0001.

Tumor growth and progression in the Rip1Tag2 model is associated with tumor angiogenesis [26, 27]. We therefore characterized the vasculature in β tumors and the exocrine pancreata of both Rip1Tag2 and Rip1Tag2CYR mice. A first visual examination of CD31-stained tumors suggested that Rip1Tag2CYR tumors were more vascularized compared to Rip1Tag2 tumors (Figure 5A). To quantify vascularization we examined four different parameters: micro-vessel density, total vessel length, area covered by vessels and the number of bifurcation points (Figure 5B). The higher values of all four parameters of Rip1Tag2CYR tumors demonstrate that tumor angiogenesis is enhanced by CYR61 expression in the β tumors.

Tumor angiogenesis in the Rip1Tag2 model largely depends on VEGF [27, 28]. To unravel whether CYR61 enhanced angiogenesis in a VEGF-dependent or independent manner, we treated 10 weeks old RipTag2 and Rip1Tag2CYR mice for 10 days with the anti-VEGFR-2 blocking antibody DC101. The enhancing effect of CYR61 on tumor angiogenesis was totally abolished by DC101 (Figure 5C). This result demonstrated that the pro-angiogenic effect of CYR61 in Rip1Tag2CYR mice fully depends on an active VEGF/VEGFR-2 pathway.

### CYR61-promoted invasiveness of β tumors is independent of loss of E-cadherin or infiltration of inflammatory cells

**Figure 6:**
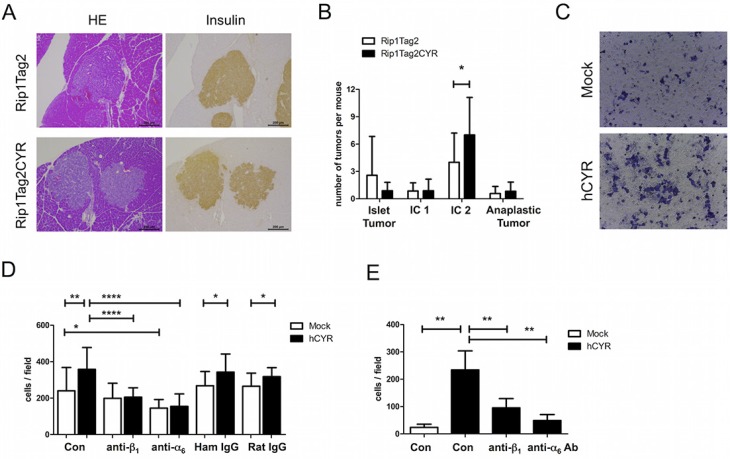
CYR61 enhances β tumor cell invasiveness via integrin α_6_β_1_ (**A**) Serial sections of β tumors with similar size from Rip1Tag2 (top row) and Rip1Tag2CYR (bottom row) mice were stained with HE and for insulin to show irregular borders and invasiveness. Scale bar: 200 μm. (**B**) Beta tumors were categorized into 4 grades according to invasiveness: *Islet Tumor*, non invasive (benign); *Invasive Carcinoma (IC1) I*, minimally invasive; *Invasive Carcinoma II (IC2)*, highly invasive; *Anaplastic Tumor*. CYR61 caused a decrease of non-invasive and an increase of highly invasive tumors. (**C**) Representative pictures showing bTC3 cell invasion *in vitro*. Mock: empty vector control. hCYR: overexpression of human CYR61. (**D**) CYR61 overexpression in bTC3 cells enhances cell adhesion on laminin. The effect was blocked by pre-incubation of anti-β_1_ or anti-α_6_ integrin antibody. Hamster IgG and Rat IgG were used as isotype control. (**E**) Treatment with anti-β_1_ and anti-α_6_ integrin antibodies significantly blocked the enhanced invasiveness of hCYR-overexpressing bTC3 cells. Isotype controls are shown in Supplementary Figure 4. Results represent mean values ± SD. *p < 0.05; **p < 0.01; ****p < 0.0001.

From histological staining, we observed that there were more irregular, invasive tumors in Rip1Tag2CYR mice compared to Rip1Tag2 mice (Figures 4A and 6A). We then classified individual tumors into 4 different categories to assess the degree of invasiveness (see Materials and Methods for the grading criteria). This classification revealed that the grading distribution of Rip1Tag2CYR tumors shifted from non- or less-invasive tumors (*Islet Tumor* and *Invasive Carcinoma I*) to more invasive tumors (*Invasive Carcinoma II*) compared to Rip1Tag2 mice (Figure 6B). The fraction of anaplastic tumors, however, remained unchanged. In addition to histological observations, *in vitro* results from invasion assay using the β tumor-derived cell line bTC3 confirmed that cell invasion was dramatically enhanced by CYR61 overexpression (Figure 6C).

In the Rip1Tag2 model, transition from adenoma to invasive carcinoma is associated with loss of E-cadherin [29], which has also been implicated in the progression of various types of human epithelial cancer and is considered as a hallmark of increased malignancy [30]. To test whether the more invasive phenotype of β tumors in Rip1Tag2CYR mice resulted from loss of E-cadherin, we stained for E-cadherin in pancreata of both Rip1Tag2 and Rip1Tag2CYR mice. The staining revealed that most of the tumors in both genotypes retained E-cadherin at cell-cell junctions (Supplementary Figure 2A a–b) and only rare relocalization from cell borders was observed throughout the tumors (Supplementary Figure 2A c–d). Furthermore, real-time PCR analysis demonstrated that the mRNA level of E-cadherin was not changed in the *ex vivo* cultured cells derived from both Rip1Tag2 and Rip1Tag2CYR tumors (Supplementary Figure 2B, right). Consistent with these results, ectopic expression of CYR61 in the β tumor-derived cell line bTC3 did not impinge on E-cadherin RNA and protein levels (Supplementary Figure 2C, 2D) or altered vimentin and N-cadherin protein expression, two markers of EMT (Supplementary Figure 2D). These results concur to indicate that CYR61-enhanced tumor invasiveness is not due to loss of E-cadherin expression.

Next, we monitored the recruitment of inflammatory cells in the tumors, as these cells can promote tumor angiogenesis, cell migration and invasion [31]. However, there was no increase in the number of infiltrating leukocytes (CD45^+^), monocytes (CD11b^+^) or M2 macrophages (F4/80^+^/MRC1^+^ or Tie2^+^) in Rip1Tag2CYR versus Rip1Tag2 tumors (data not shown).

### CYR61 promotes invasiveness of β tumors through cooperation with integrin α_6_β_1_

The observation of α cell segregation suggests that the function of integrins might be affected by ectopic CYR61 expression in β cells. To test this hypothesis, we used bTC3 cells with or without CYR61 overexpression to examine the expression and function of integrins. Flow cytometry analyses showed that CYR61-overexpression in bTC3 cells did not alter surface expression level of α_v_, α_4_, α_6_, β_1_, β_3_ and β_4_ integrin subunits. Since β_1_ and α_6_ are the most abundantly expressed subunits (Supplementary Figure 3A), we performed an adhesion assay on the α_6_β_1_ ligand laminin. CYR61 expression significantly enhanced cell adhesion to laminin, which was blocked by pre-incubation with either β_1_ or α_6_ antibody (Figure 6D). Moreover, the same antibody treatment blocked the enhanced invasion induced by CYR61-overexpression (Figure 6E). Collectively, these data indicate that CYR61-induced increased β cell invasiveness is mediated by augmented integrin α_6_β_1_ function.

## DISCUSSION

In this paper we report the first characterization of transgenic mice expressing human CYR61 in the pancreatic β cells under normal (Rip1CYR) and tumorigenic (Rip1Tag2CYR) conditions. While using human CYR61 to generate transgenic mice could raise potential concerns about its biological activity across species, we believe that this will be unlikely the case. CYR61 protein is highly conserved across human and mouse. They share 92.8% sequence identity and differ in length by only 2 amino acids (2 more in human). The functional domains are 100% homologous and most of the variable amino acids are located in the linker region between the VWC and TSP-1 domains [32]. Therefore, we expect the human CYR61 protein to retain its functional properties in the mouse background environment.

Rip1CYR mice breed normally and show only a mild phenotype: abnormal sorting of α cells and altered morphology of Langerhans' islets. During mouse pancreas development, segregation of α cells toward the islets' periphery begins at 17.5–18 days *post coitum* and continues until 4–5 weeks of age [33]. Histological staining of α cells in 13-week old Rip1CYR mice revealed that about 30% of the islets have abnormal α cell distribution. The same distorted phenotype was reported in β cell-specific transgenic mice with deficiency of integrin β_1_, N-CAM or with impaired E-cadherin function [34–36]. In our model, CYR61-overexpressing bTC3 cells showed increased cell adhesion and migration capacities (Figure 6D–6E) suggesting that the activity of β_1_ and α_6_ integrins might be enhanced in the β cells of Rip1CYR mice. Therefore, we hypothesize that positively or negatively altering the adhesive balance of β cells might affect the interaction between α and β cells resulting in perturbed α cell guidance to or retention at the islets periphery. Further experiments are needed to unravel how exactly enhanced CYR61 expression in β cells affect sorting of α cells.

On the other hand, the enhanced integrin activity is consistent with our observations of irregular and invasive morphologies in islets of Rip1CYR mice and β tumors of Rip1Tag2CYR mice. Moreover, the presence of larger tumors in the Rip1Tag2CYR mice (Figure 4) further supports the notions of enhanced integrin function by CYR61 expression, since β tumors lacking integrin β_1_ are smaller than control tumors [34]. Therefore, we conclude that integrin gain-of-function is involved in the invasive phenotypes observed in islets and tumors in Rip1CYR and Rip1Tag2CYR mice, respectively.

Interestingly, transgenic expression of CYR61 in Rip1CYR mice did not induce β cell hyperplasia or β tumor formation (mice were monitored up to 18 months) even though CYR61 expression has been linked to enhanced cell proliferation and tumorigenesis [4]. Rip1Tag2CYR mice, however, have larger tumors compared to Rip1Tag2 mice. These observations indicate a tumor promoting rather then a tumor initiating function of CYR61. CYR61 expression is insufficient to bypass intact cell cycle checkpoints to initiate β cell proliferation. Only when cell cycle checkpoints are compromised, as it occurs in Rip1Tag2 tumors through the inhibition of p53 and Rb functions by the large T antigen, CYR61 has a tumor promoting effect resulting in a higher tumor burden.

Besides tumor size, a similar ancillary effect of CYR61 was observed on tumor angiogenesis. Previous studies have implicated CYR61 as a major physiological mediator of vascular development. This conclusion was mainly based on *in vitro* studies on endothelial cells [7] and *in vivo* studies using *Cyr61-*null mice [25]. However, the detailed mechanism of how CYR61 modulates vascular formation *in vivo* is still unclear. We found that transgenic expression of CYR61 did not alter vasculature in the islets of Rip1CYR mice, but was able to enhance tumor-induced angiogenesis in Rip1Tag2CYR mice. Since tumor angiogenesis in Rip1Tag2 model is mainly dependent on the VEGF pathways [27, 28], we treated Rip1Tag2 and Rip1Tag2CYR mice with DC101 to block VEGF/VEGFR-2 signaling. DC101 treatment suppressed angiogenesis in Rip1Tag2CYR mice to the same extent as in Rip1Tag2 mice, indicating that the angiogenic activity of CYR61 was dependent on the VEGF/VEGFR-2 axis. While our findings do not dispute the essential role of CYR61 in vascular development obtained from gene deletion studies, they clearly indicate that CYR61 itself as an ancillary role in promoting angiogenesis: CYR61 expression alone is not sufficient to induce *de novo* angiogenesis (i.e. in Rip1CYR mice). However, when angiogenesis is already induced, as it occurs in hyperplastic islets and tumors in Rip1Tag2CYR mice, ectopic CYR61 expression further enhances it.

In conclusion, our results provide the first evidence that CYR61 alters the architecture (β cell protrusion) and affects α cell sorting in normal islets in Rip1CYR mice, while it promotes growth and invasion of insulinoma in the Rip1Tag2 model. These effects might be due to increased activity of integrin α_6_β_1_. Ectopic expression of CYR61 does not initiate angiogenesis in quiescent islets but enhances VEGF-dependent tumor angiogenesis in insulinoma. Taken together, these observations demonstrate that CYR61 acts as a tumor-promoting gene in pancreatic neuroendocrine tumors.

## MATERIALS AND METHODS

### Transgenic mouse lines

Animal experiments were approved by the Cantonal Office in Fribourg (FR_2011_34_FR) and performed according to federal regulations. Rip1CYR transgenic mice were generated following standard procedures [37]. The transgene was constructed by cloning a 1620 bp cDNA fragment containing the human *CYR61* ORF (1146 bp) between the 695-bp Bam HI/XbaI fragment of the rat insulin gene II promoter and a 436-bp DNA fragment containing the SV40 small t antigen intron and polyadenylation signal. PCR primers for Rip1CYR heterozygotes genotyping were: CYR-F: 5′-GTCTCAGTCGAGGTGAGGACACAG-3′. CYR-R: 5′-GATGCGGGAGCTCATTGTGGC-3′. Amplicon: 650 bp. PCR cycles were: 95°C, 5 min (1x); 95°C, 30 sec, 60°C, 30 sec, 72°C, 1 min (35x); and 72°C, 5 min (1x). Generation and phenotypic characterization of Rip1Tag2 mice was performed as described previously [24]. PCR primers for Rip1Tag2 heterozygotes genotyping were: Tag2-F: 5′-GGACAAACCACAACTAGAATGCAG-3′. Reverse: 5′-CAGAGCAGAATTGTGGAGTGG-3′. Amplicon: 500 bp, PCR cycles were: 95°C, 5 min (1x); 94°C, 30 sec, 53°C, 30 sec, 72°C, 40 sec (35x); and 72°C, 10 min (1x). Both Rip1CYR and Rip1Tag2 mice were kept in the C57Bl/6 background. Rip1CYR transgenic mice were maintained for up to 18 months while Rip1Tag2, and Rip1Tag2CYR mice were maintained until 14 weeks of age.

### *In vivo* anti-VEGFR2 treatment

For anti-VEGFR-2 treatment, 10-week old Rip1Tag2 and Rip1Tag2CYR mice were randomly grouped and injected with 1 μg of DC101 (BioXCell, West Lebanon, NH, #BE0060) or isotype control IgG (BioXCell, #BE0088) at Day 1, 5, 8. Mice were sacrificed at Day 10.

### Immunohistochemical staining

Tissue sections were heated in Tris-EDTA buffer to retrieve antigen epitopes, blocked by 10% normal goat serum and Avidin/Biotin blocking reagent (Vector Laboratories, Burlingame, CA) and stained with the following primary antibodies at 4°C overnight: anti-insulin (DAKO, Baar, Switzerland, #A0564), anti-CYR61 (Santa Cruz Biotechnology, Inc., Heidelberg, Germany, #sc13100), anti-glucagon (DAKO, #A0565), anti-CD31 (Neomarkers Inc, Fremont, CA, #RB-10333-P), anti-E-cadherin (BD Biosciences, Basel, Switzerland, #610182). Sections were incubated with biotinylated secondary antibodies followed by Vectastain ABC Kit (Vector Laboratories). DAB peroxidase substrate (Sigma-Aldrich, Buchs, Switzerland) was used to reveal the signal from antibody-peroxidase complex. Sections were counterstained with hematoxylin before mounting.

### Image analysis

### Size of islets of Langerhans and β tumors

H&E stained sections were scanned and the area of islets or tumors were determined using Image J software (NIH, Bethesda, MD). The average size of islets obtained from wild type and Rip1CYR mice were used to exclude normal islets in the pancreata of Rip1Tag2 and Rip1Tag2CYR mice.

### Tumor grading

*Islet Tumor*: tumors with no invasive protrusion; *Invasive Carcinoma I*: tumors with one invasive front and residual epithelial organization; *Invasive Carcinoma II*: tumors with multiple (i.e. > 2) invasive fronts and highly invasive morphology; and *Anaplastic Tumor*, tumors with atypical nuclei and high nucleus/cytoplasm ratio.

### Vasculature parameters

Tissue sections were stained with endothelial cell marker CD31. Chalkley grid [38] was used to evaluate micro-vessel density in β tumors (Figure 5B). In β islets (Figure 3B) and β tumors with DC101 treatment (Figure 5C), micro-vessel density was determined by normalizing vessel numbers with area. Total length of vessels, area covered by vessels and the number of bifurcation points, were determined using Image J.

### Western blot

Whole pancreata were lysed in RIPA buffer by TissueLyser LT (Qiagen, Hilden, Germany). Lysates were loaded to SDS-polyacrylamide gels and transferred to Immobilon-P membranes (Millipore, Billerica, MA). Blots were sequentially incubated with 5% BSA, primary antibody (CYR61: Santa Cruz Biotechnology; GAPDH: Sigma-Aldrich; E-cadherin and N-cadherin: BD Biosciences; Vimentin: DAKO), HRP-labeled secondary antibody (DAKO). Signals were revealed with Luminata Western HRP Substrate (Millipore, Schaffhausen, Switzerland) and detected with X-Ray films.

### Real time RT-PCR

Whole pancreata were lysed in TriPure Isolation Reagent (Roche, Mannheim, Germany) by using TissueLyser LT. Total RNA was isolated according to manufacturer's instruction. For cDNA synthesis, up to 5 mg of total RNA was mixed with Superscript First Strand Synthesis Kit (Invitrogen, Life Technologies, Basel, Switzerland). Real-time PCR was done by using KAPA SYBR FAST Universal 2x qPCR Master Mix kit (Kapa Biosystems, Boston, MA) on the Step One Plus Real-Time PCR System (Applied Biosystems, Life Technologies). Dissociation curve of each reaction was checked to determine the purity of the PCR products. Relative Ct values of target genes to housekeeping gene (36B4) were calculated to compare the expression level between samples. All PCR reactions were done in triplicate. Primer sequences are as following: Human CYR61: Forward: 5′-ACGCTGGATGTTTGAGTGTG-3′. Reverse: 5′-TGTAGAAGGGAAACGCTGCT-3′. Mouse CYR61: Forward: 5′-GAACCGCGAGTTCTTTTCAA-3′. Reverse: 5′-AGGACGCACTTCACAGATCC-3′. Mouse E-cadherin: Forward: 5′-CAAGGACAGCCTTCTTTTCG-3′. Reverse: 5′-TGGACTTCAGCGTCACTTTG-3′. Mouse 36B4: Forward: 5′-GTGTGTCTGCAGATCGGGTAC-3′. Reverse: 5′-CAGATGGATCAGCCAGGAAG-3′.

### Generation of *ex vivo* cultured β tumor cell lines

Beta tumors were excised from pancreas, minced into small pieces, transferred into tubes with medium and let stand for 1 minute for the bigger tissue fragments to pellet. Supernatant was then transferred to new tubes and let stand for 15 minutes. Finally, supernatant and pellet were carefully separated and serially diluted into individual wells of a 6-well plate. DMEM medium with 10% FBS was changed every 2–3 days.

### Overexpression of CYR61 in the bTC3 cell line

The bTC3 cell line, provided by Dr. Hanahan [27], was maintained in DMEM with 10% FBS and sub-cultured every 3–4 days. CYR61 overexpression was achieved through lentiviral transduction. Cells were incubated with lentivirus-containing supernatant supplemented with 10% complete DMEM and 8 μg/mL polybrene for 6 hours. Afterwards, medium was replaced with fresh culture medium and the cells were kept for 2 days before puromycin selection.

### Flow cytometry

Cells were trypsinized, recovered in serum-free medium at 37°C for 1 hour, and blocked with anti-mouse CD16/CD32 antibodies (BD Biosciences). Fluorochrome-conjugated integrin antibodies were diluted 1:50 and the staining was performed on ice for 30 minutes. Cells were washed twice and applied to FACSCalibur (Becton Dickinson, Mountain View, CA). Results were analyzed using FlowJo software (Ashland, OR).

### Invasion assay

A modified flow-driven transwell migration system was adopted as previously described [39]. Cells mixed with 1.2 mg/mL of Rat Tail Collagen I (BD Biosciences) and 10% growth factor-reduced matrigel (BD Biosciences) were placed to the upper part of Transwell inserts (8 μm pore size, BD Biosciences) and incubated at 37°C for 1 hour to solidify the cell-matrix mixture. 150 μL serum-free DMEM was loaded to the lower part of the chamber. 650 μL serum-free DMEM was added on top of the gel to create a hydrostatic pressure mimicking interstitial flow. After overnight incubation, non-migrated cells and the gel were carefully removed with cotton swabs. Migrated cells on the other side were fixed with 4% PFA and stained by crystal violet. Five fields per insert were photographed to cover the vast area of whole insert. The representative data from one of the three independent experiments are shown.

### Adhesion assay

Coverslips were coated with 10 μg/mL of laminin at 37°C for 3 hours, followed by 1 hour of blocking at 37°C with 1% BSA. Cells re-suspended in serum-free medium were blocked with anti-mouse CD16/CD32 antibodies (BD Biosciences, #553142) and then treated with 5 μg/mL of integrin blocking antibodies (β_1_ integrin: Biolegend, San Diego, CA, #102201; α_6_ integrin: Biolegend, #313602) or isotype control IgG (Hamster IgG: Biolegend, #400901; Rat IgG: Biolegend, #400501) for 15 minutes before seeding on laminin-coated coverslips. After 1 hour of adhesion at 37°C, unattached cells were washed away with PBS. Adhered cells were fixed by 4% PFA and random fields were chosen to take pictures for quantification. Representative results from three independent experiments are shown.

### Statistical analysis

Results were expressed as mean ± SD. For most experiments, the statistical validation was performed by Mann-Whitney test. Two-way ANOVA was used to analyze the results of tumor grading (Figure 4B). *P*-value < 0.05 was considered statistically significant. *p < 0.05; **p < 0.01; ***p < 0.001; ****p < 0.0001.

## SUPPLEMENTARY MATERIALS FIGURES


