# Supplementary material for: The matricellular protein CYR61 interferes with normal pancreatic islets architecture and promotes pancreatic neuroendocrine tumor progression
Source: Oncotarget. 2015 Nov 27;7(2):1663–74. doi: 10.18632/oncotarget.6411 (PMC4811488; doi:10.18632/oncotarget.6411)
Supplement: Supplementary file 1 [file oncotarget-07-1663-s001.pdf]

The matricellular protein CYR61 interferes with normal pancreatic islets architecture and promotes pancreatic neuroendocrine tumor progression

Supplementary Materials

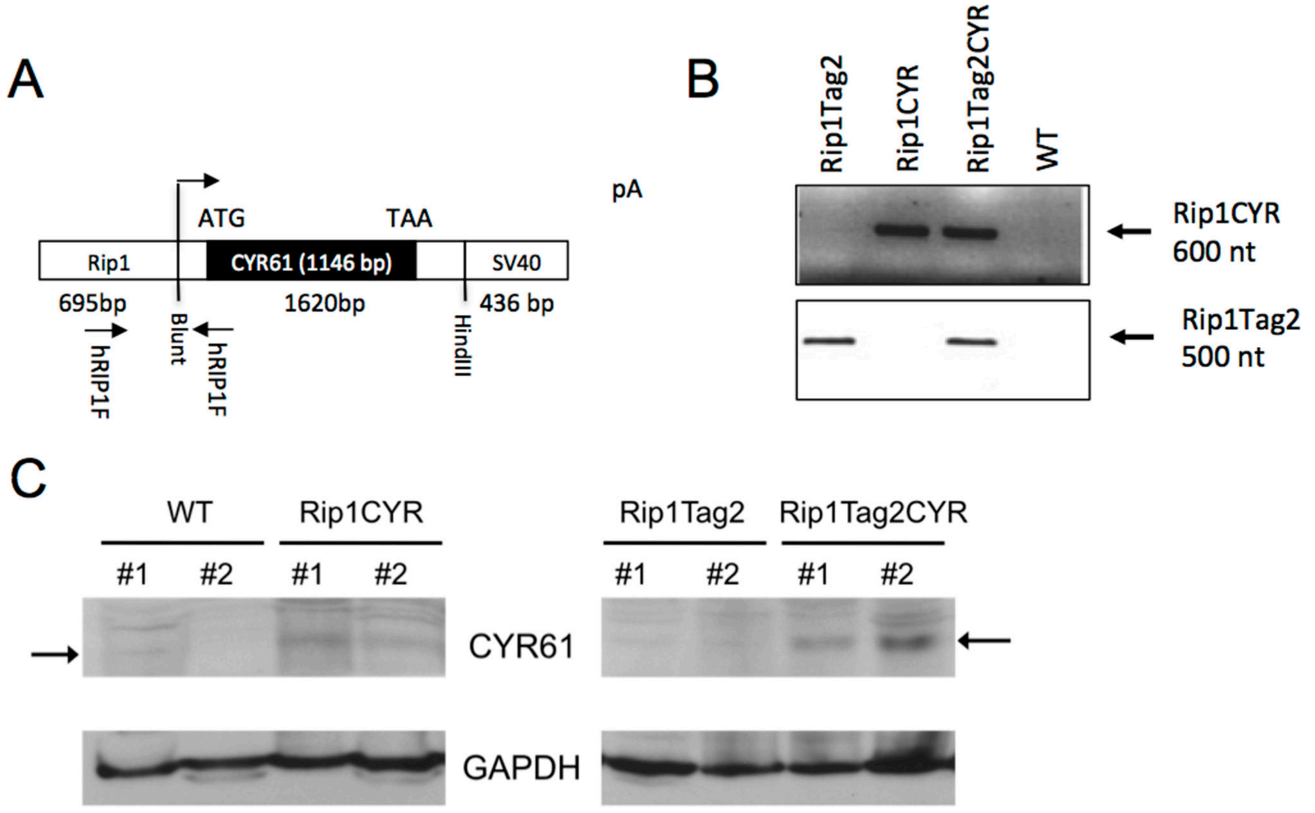

Supplementary Figure S1: Transgene construct and CYR61 transgene expression in Rip1CYR mice. (**A**) Schematic description of the transgene construct, which contains CYR61 cDNA under the control of rat insulin promoter 1 (Rip1). (**B**) Representative result of genotyping PCR showing the expression of CYR61 and SV40 T antigen. The genotype is indicated on top of the lanes. The DNA products amplified by specific primers and amplicon size are shown on the side. (**C**) Western blot analysis of CYR61 from whole pancreatic tissue lysates demonstrating transgene expression. The arrows highlight the position of CYR61 bands.

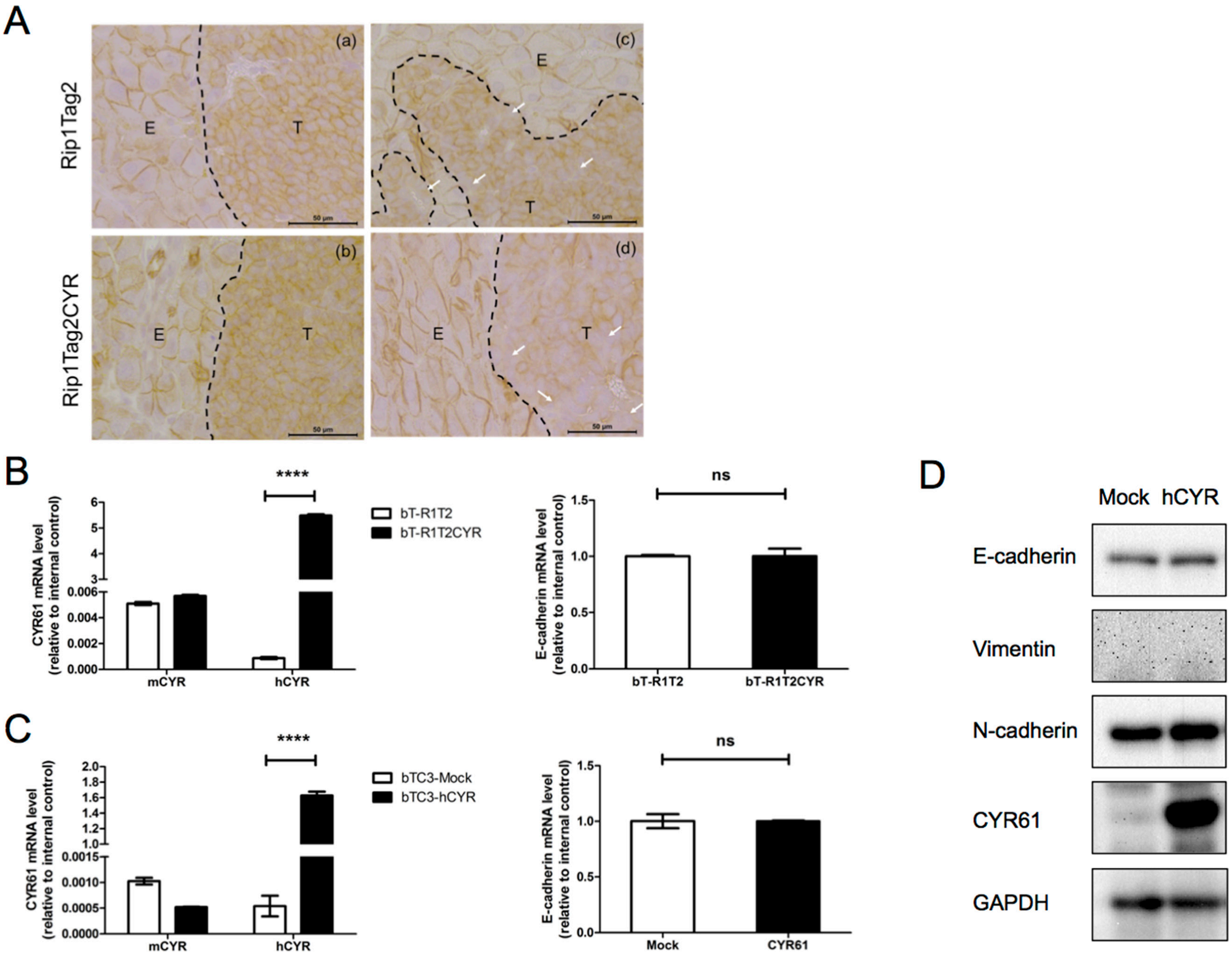

Supplementary Figure S2: CYR61 transgenic expression has only minor effect on E-cadherin expression. (**A**) E-cadherin staining of β tumors from Rip1Tag2 and Rip1Tag2CYR mice. E-cadherin expression at cell-cell junctions was observed in most tumors of both genotypes (a, b). Partial dislocation of E-cadherin from cell borders was more obvious in the Rip1Tag2CYR tumors (c, d). White arrows: partial loss of E-cadherin. E, exocrine pancreas; T, tumor. Scale bar: 50 µm. (**B**) Measurement of E-cadherin mRNA by real-time PCR in *ex vivo* cultured cell lines derived from Rip1Tag2 (bT-R1T2) and Rip1Tag2CYR (bT-R1T2CYR) tumors. Rip1Tag2- and Rip1Tag2CYR-derived lines express comparable levels of E-cadherin mRNA. (**C**) Measurement of E-cadherin mRNA by real-time PCR in the β tumor-derived cell line bTC3 (Mock: empty vector control) and in bTC3 overexpressing CYR61. Overexpression of CYR61 in bTC3 cells had no effect on the mRNA level of E-cadherin. Mock: empty vector control. (**D**) Comparable protein levels of E-cadherin, N-cadherin and Vimentin were detected in Mock control and CYR61-overexpressing bTC3 cells. Results represent mean values ± SD. ns: non significant; ****$p < 0.0001$.

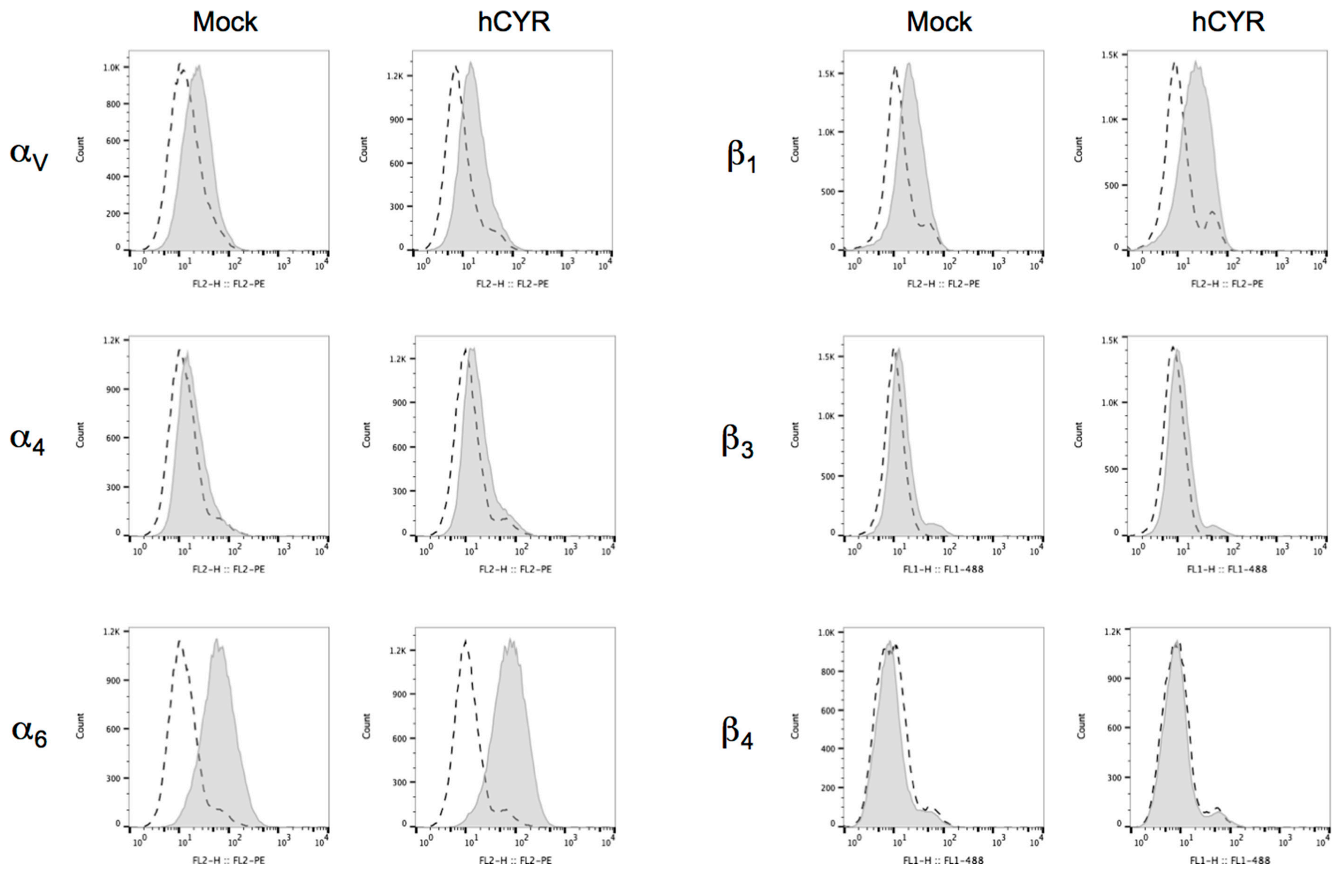

Supplementary Figure S3: CYR61 overexpression does not alter surface levels of integrin subunits expression in bTC3 cells. Expression levels of integrin α_v, α_4, α_6, β_1, β_3, and β_4 on the cell surface of bTC3 cells with (hCYR) or without (Mock) CYR61 overexpression were monitored by flow cytometry. Gray histograms represent the signal from indicated integrin subunit, and the dashed histograms denote the corresponding IgG isotype control antibodies.

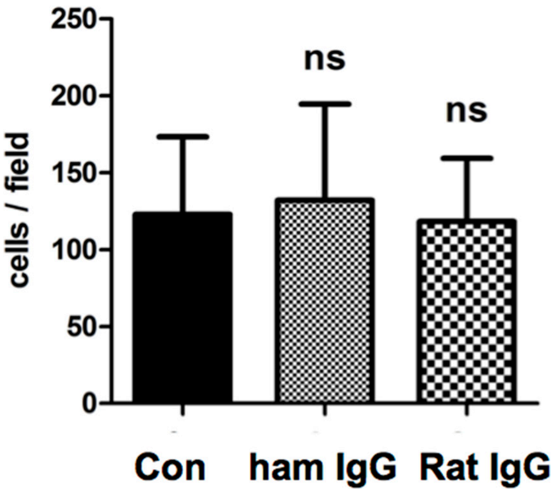

Supplementary Figure S4: The IgG isotype control antibodies do not affect invasion of bTC3-hCYR cells. CYR61-overexpressing bTC3 cells were treated with the isotype control antibodies for anti-β_1 (hamster IgG and anti-α_6 (Rat IgG) antibodies and applied to the invasion assay. Control antibodies had no effect on bTC3 cell invasion. Results represent mean values ± SD. ns: non significant.
